# Supplementary material for: Chemical and genomic characterization of a potential probiotic treatment for stony coral tissue loss disease
Source: Commun Biol. 2023 Apr 6;6:248. doi: 10.1038/s42003-023-04590-y (PMC10079959; doi:10.1038/s42003-023-04590-y)
Supplement: Supplementary file 2 — Supplementary Information [file 42003_2023_4590_MOESM2_ESM.pdf]

# Supplementary Materials for

## Chemical and genomic characterization of the first potential probiotic treatment for a coral disease threatening the Caribbean

Blake Ushijima*, Sarath P. Gunasekera, Julie L. Meyer, Jessica Tittl, Kelly A. Pitts, Sharon Thompson, Jennifer M. Sneed, Yousong Ding, Manyun Chen, L. Jay Houk, Greta S. Aeby, Claudia C. Häse, Valerie J. Paul*

*Blake Ushijima, ushijimab@uncw.edu; Valerie Paul, paul@si.edu

**This PDF file includes:**

Supplementary Note
Supplementary Methods
Supplementary Figs. S1 to S16
Tables S1 to S6
References (7, 74-79)

**Supplementary Note**

*A previously unknown biosynthetic gene cluster for korormicin was putatively identified*

In strain McH1-7, the biosynthesis of korormicin A is predicted to include the formation of the nonproteinogenic amino acid (2*S*)-2-$NH_2$-4-OH-4-methylhexanoate (**Figure 2**). A set of six enzymes Kor16-21, whose genes seem to form a single operon, may sequentially convert 2-oxoisoleucine, the immediate precursor and catabolic product of L-isoleucine, into (2*S*)-2-$NH_2$-4-OH-4-methylhexanoate in a process analogous to the biosynthesis of L-leucine from 2-oxovaline (**Figure 2**). Indeed, these enzymes are predicted to be 2-isopropylmalate synthase (LeuA, Kor21), the small and large subunits of 3-isopropylmalate dehydratase (LeuCD, Kor16, Kor17 and Kor20), and 3-isopropylmalate dehydrogenase (LeuB, Kor18). For McH1-7, the installation of the α-amino group appears to be catalyzed by the pathway-specific Kor1, a predicted class III aminotransferase, instead of the branched-chain amino acid transaminase IlvE utilized for amino acid biosynthesis (**Figure 2**) [63]. Interestingly, the *kor* cluster encodes two 3-isopropylmalate dehydratase large subunit homologs Kor17 and Kor20, while only one (LeuC) is required for the biosynthesis of L-leucine. Kor17 and Kor20 share 51% amino acid identities and carry three conserved cysteine residues for the binding of the cofactor [4Fe-4S] cluster [64]. Phylogenetic analysis revealed that Kor17, Kor20 and LeuC homologs form three clades (**Figure S10**), indicating separate evolution events. Of note, the *kor* gene cluster encodes a putative iron-sulfur cluster insertion protein (Kor5) that may facilitate the maturation of Kor17 and Kor22. One putative α-ketoglutarate-dependent dioxygenase Kor19 in the operon may hydroxylate the α-amino acid intermediate to produce (2*S*)-2-$NH_2$-4-OH-4-methylhexanoate as the substrate of one predicted nonribosomal peptide synthetase (NRPS) Kor26 that is comprised of one condensation (C), one adenylation (A), one thiolation (T) and one thioester (TE) domain (**Figure 2**)[65]. The A domain of Kor26 is predicted to activate hydrophobic amino acid building blocks.

The *kor* gene cluster encodes three putative single-module polyketide synthases (PKSs, Kor23-25) and one loading module (Kor22) (**Figure 2**). Kor22 contains one fatty acid AMP-ligase (FAAL), one acyl-CoA dehydrogenase (DeH) and one T domain (**Table 1**). The FAAL likely selects lauric acid (12-C) as substrate for the biosynthesis of korormicin analogs with an 18-C polyketide tail, while fatty acids with different chain lengths may serve as the starter units of other korormicin analogs, e.g., decanoic acid for korormicin B [28,32]. The DeH domain of Kor22 shares a high similarity with many standalone acyl-CoA dehydrogenases and carries one strictly conserved, catalytically essential Glu [66]. Acyl-CoA dehydrogenases generally produce α,β-unsaturated acyl-CoAs from acyl-CoA substrates, which is unlikely relevant to the biosynthesis of korormicins. Kor23-25 together are expected to extend the fatty acid starter unit by six carbons using three molecules of malonyl-CoA (M-CoA, **Figure 2**). Kor23 and Kor25 are comprised of one ketosynthase (KS), one uncharacterized domain, one ketoreductase (KR) and T domain in order (**Table 1**). The uncharacterized domain of Kor23 and Kor25 is about 600 amino acids in size, and further bioinformatics analysis revealed their *N*-terminal region of about 400 amino acids shares similarities with the acyltransferase (AT) domain of several uncharacterized cyanobacterial PKSs (e.g., 42% similarities with the PKS with Genbank accession number of WP_106919628.1). We thus denoted this domain as AT* and proposed it to be functional and select M-CoA as substrate, while it remains unexcluded that the AT domains of other PKS clusters may contribute to the korormicin biosynthesis. The domain organization of Kor24 is KS-

AT-dehydratases (DH)-KR-T (**Table 1**). The KR-DH didomain of Kor24 likely forms a *cis*-double bond, similar to those in the biosynthesis of phoslactomycin [67]. In addition, as either Kor22 or Kor25 lacks a DH domain, it might be possible that the DH domain of Kor24 may act with the KR domain of the preceding PKS to produce a *trans*-double bond observed in the final structures of korormicins (**Figure 2**). This hypothesis awaits further tests. The KR domain of Kor25 is predicted to produce an A-type hydroxyl group [68], while the stereochemistry of the KR domain of Kor22 is bioinformatically undefinable. Since all korormicin analogs carry a 3'-*R*-OH group at their acyl chains, Kor25 may catalyze the last polyketide chain extension.

Next, the C domain of Kor26 likely catalyzes the formation of one peptide bond between the final polyketide intermediate tethered to Kor25 and the loaded (2*S*)-2-$NH_2$-4-OH-4-methylhexanoate (**Figure 2**). The TE domain then releases the hybrid intermediate by forming one γ-lactone, which is then converted into korormicin K by forming a 9'-10' double bond. Kor27 can be the responsive $\Delta^9$ fatty acid desaturase. Alternatively, the DeH* domain of Kor22 may perform an uncommon β,γ-dehydrogenation on the loaded fatty acid starting unit (**Figure 2**). Korormicin K is further converted into korormicin G after the epoxidation of the 9'-10' double bond, likely mediated by the second pathway-specific α-ketoglutarate-dependent dioxygenase Kor13. In korormicin F, H, and I, this epoxide is opened by the attack of $Br^-$ or $Cl^-$. Finally, korormicin A is produced after forming one double bond on C3 and C4, as observed in other analogs (e.g., korormicin B, C, D and E). Kor15 is a predicted trans-2-enoyl-CoA reductase and might catalyze this dehydrogenation reaction (**Table 1**).

The *kor* gene cluster encodes gene products for regulation and resistance to korormicin production. In particular, *kor28-36* seems to form an operon that encodes all six subunits of NADH:ubiquinone reductase (NQR, Kor28-33) and two auxiliary proteins, FAD:protein FMN transferase (Kor34) and ($Na^+$)-NQR maturation NqrM (Kor36). Korormicins bind to the subunit B of NQR (NqrB) to execute their bioactivities [30]. Importantly, Kor29 encoding NqrB carries a critical Gly141-to-Ala mutation, which is known to provide resistance of *Pseudoalteromonas* sp. J010 to korormicins [31,69]. Furthermore, the *kor* gene cluster encodes one putative major facilitator superfamily (MFS) transporter (Kor9) that can export korormicins. The pathway-specific regulators include one helix-turn-helix transcriptional regulator (Kor8) and one BolA family transcriptional regulator (Kor14). In addition, the *kor* gene cluster carries nine genes whose encoded products remain uncharacterized and require additional research to investigate their contributions to the biosynthesis of korormicins.

*McH1-7 is detectable at low levels over 28 days after inoculation in healthy corals*

Gene copies of the Kor23 gene of korormicin biosynthetic gene cluster were detected by ddPCR in most aquaria water samples, including samples from one hour after inoculation, and 1 day, 3 days, 7 days, 21 days, and 28 days post-inoculation (**Figure 6**). The addition of McH1-7 is clearly reflected as an increase of detected korormicin Kor23 gene copies (**Figure S11**). Only the tank water holding coral genotype 25 had detectable korormicin Kor23 gene copies at every time point and this tank also had the highest gene copies at day 1 of any sample or time point in the study (**Figure S11**). Overall, korormicin Kor23 gene copies were detected at lower levels in the tissue samples compared to water samples (**Figure 6**). Korormicin gene copies were not detected in any tissue samples prior to inoculation with McH1-7 nor on day 3 post-inoculation. The highest detected level of korormicin gene copies was on day 7 post-inoculation, in coral genotype 26. Korormicin gene copies were detected in tissue samples from coral genotypes 25

and 26 on day 21 post-inoculation and only in coral genotype 25 on day 28 post-inoculation (**Figure 6**). Overall, these results suggests that McH1-7 is not maintained at relatively high concentrations over time on coral fragments.

Microbial community composition among the 24 tissue/mucus samples was variable both within and among coral fragments (**Figure S12**). Microbiome composition did not vary by coral genotype (PERMANOVA $R^2 = 0.31074$, p = 1.0), by date (PERMANOVA $R^2 = 0.13516$, p = 1.0), nor by a combination of genotype and date (PERMANOVA $R^2 = 0.55409$, p = 1.0). This is reflected in the lack of clustering in the principal component analysis and in the variability of community structure at the class level (**Figure S12**), where there is as much variation within fragments from the same original coral colony as there is between fragments of different colonies. The lack of significant patterns may be the result of the relatively small sample size (n=24) or because each time point was from a different coral fragment, suggesting either the microbiome is heterogeneous across colonies or that the communities shifted after fragmentation. These factors versus the effects of inoculation with McH1-7 cannot be teased apart with the current dataset, however, these results show that McH1-7 does not take over the coral microbiome after inoculation of the water column.

Only four *Pseudoalteromonas* amplicon sequence variants were detected in the 16S amplicon libraries. One of these 253-bp amplicon sequence variants is an exact match to the V4 region of the 16S rRNA gene from the *Pseudoalteromonas* sp. McH1-7 genome and it was detected just once, in coral genotype 28 on day 7 post-inoculation. The sensitivity of the 16S amplicon libraries in detecting *Pseudoalteromonas* amplicon sequence variants was similar to the korormicin ddPCR results on the same DNA extracts. The partial korormicin gene was detected in six tissue/mucus samples and *Pseudoalteromonas* amplicon sequence variants were detected in five tissue/mucus samples. Only one sample (coral genotype 25 on day 28 post-inoculation) showed the presence of both the korormicin gene and a *Pseudoalteromonas* amplicon sequence variant.

## Supplementary Methods

*Chemical analysis*

The optical rotations were recorded on a Rudolph Research Analytical Autopol III automatic polarimeter. UV spectrophotometric data was acquired on a Shimadzu PharmaSpec UV-visible spectrophotometer. NMR data were collected on a JEOL ECA-600 spectrometer operating at 600.17 MHz for $^1$H and 150.9 MHz for $^{13}$C. The edited-HSQC experiment was optimized for $J_{CH} = 140$ Hz and the HMBC spectrum was optimized for $^{2/3}J_{CH} = 8$ Hz. $^1$H NMR chemical shifts (referenced to residual $CHCl_3$ observed at $\delta$ 7.25 were assigned using a combination of data from 2D DQF COSY and multiplicity-edited HSQC experiments. $^1$H NMR chemical shifts for residual DMSO-$d_6$ observed at $\delta$ 2.49. Similarly, $^{13}$C NMR chemical shifts (referenced to $CDCl_3$ observed at $\delta$ 77.0) were assigned on the basis of multiplicity-edited HSQC experiments. The HRMS data were obtained using an Agilent 6210 LC-TOF mass spectrometer equipped with an APCI/ESI multimode ion source detector at the Mass Spectrometer Facility at the University of California, Riverside, California. Silica gel 60 (EMD Chemicals, Inc. 230-400 mesh) was used for column chromatography. All solvents used were of HPLC grade (Fisher Scientific).

*Isolation and structure determination*

The freeze-dried biomass (STMcH1-7) was extracted with MeOH – EtOAc (1:1). This non-polar extract on bioassay-guided reversed phase column chromatography followed by reversed phase HPLC furnished bioactive compound **1** as colorless oil. HRESI/APCIMS gave the molecular formula of $C_{25}H_{39}NO_5$. The interpretation of DQF COSY, edited HSQC and HMBC experiments (Table S2a), showed the $^1$H and $^{13}$C NMR signals of **1** were assignable to two primary methyl groups C-6, [$\delta_H$ 0.88 (t, $J$ = 6.9 Hz), $\delta_C$ 8.2) and C-18' [$\delta_H$ 0.86 (t, $J$ = 7.3 Hz), $\delta_C$ 14.1), one isolated methyl C-7 ($\delta_H$ 1.47, $\delta_C$ 24.2), seven consecutive methylenes, three isolated methylenes. There were three oxymethines of which two are coupled to each other C-9' ($\delta_H$ 2.98, $J$ = 4.2 Hz; $\delta_C$ 55.8) and H-10' ($\delta_H$ 2.95, $J$ = 4.2 Hz; $\delta_C$ 57.1) and their $^{13}$C values and coupling constants indicated the presence of an epoxide ring. Four conjugated olefinic methines, one tertiary olefinic carbon bonded to a hetero atom C-2 ($\delta_C$ 126.7), one tertiary sp3 carbon bonded to an oxygen atom C-4 ($\delta_C$ 88.4), two ester of amide type carbonyl groups C-1 ($\delta_C$ 169.2) and C-1' ($\delta_C$ 170.2). The COSY data analysis indicated in dark lines identified the ethyl group C-5 to C-6 (Figure S1a) and C-2' to C-18' carbon chain (Figure S1b). The combination of COSY correlation data indicated in dark lines and HMBC correlation data indicated in curved arrows shown in partial structures established the two partial fragments of 4-hydroxy-2- amino acid and a trihydroxy unsaturated fatty acid in the molecule.

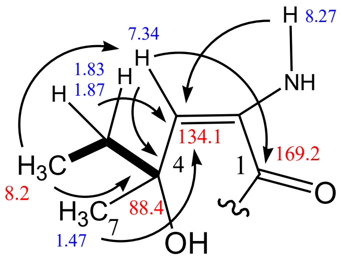


Figure 1a

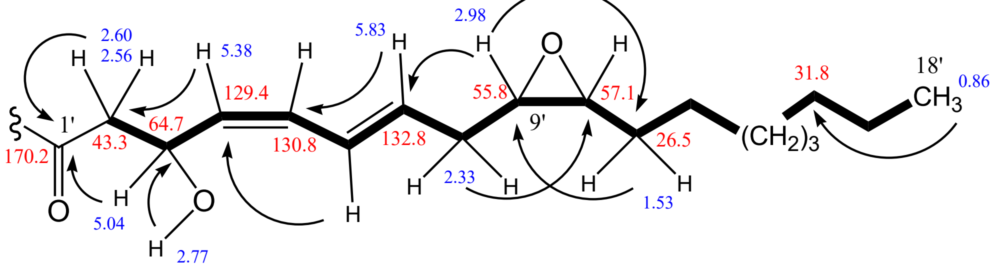


Figure 1b

**Supplementary Figure S1**. Correlated Spectroscopy (COSY) data analysis of korormicin isolated from McH1-7 cultures.

This information also established the positions of the conjugated double bonds and the position of the epoxide ring in the fatty acid chain. The formation of a $\gamma$-lactone ring satisfied the $^{13}$C values observed C-4 ($\delta_C$ 88.4), C-1 ($\delta_C$ 169.2) and the remaining unaccounted one degree of

unsaturation. HMBC correlations from 2-NH ($\delta_H$ 8.27, s) to C-1' ($\delta_C$ 170.2) and the 2DNOE data observed from 2-NH ($\delta_H$ 8.27, s) to 2'-Ha, Hb ($\delta_H$ 2.56 and 2.60) connected the γ-lactone moiety to the $C_{18}$ acid chain forming the amide bond in the molecule. The observed coupling constants of 10.9 Hz and 15.1 Hz for $J_{4',5'}$ and $J_{6',7'}$ respectively, indicated the *Z*- and *E*-configurations for each double bond. The vicinal coupling constant of 4.2 Hz for $J_{9',10'}$ and the presence of NOESY correlations between C-8' ($\delta_H$ 2.33) and C-9' ($\delta_H$ 2.51-2.53) implied that the epoxide has the *cis* orientation. De-replication using the molecular formula of $C_{25}H_{39}NO_5$ in the marine natural product database together with the described $^1$H and $^{13}$C NMR spectral analyses identified the isolate **1** as the korormicin reported in the literature.[24] The $^1$H NMR data of **1** and korormicin in DMSO-$d_6$ reported in the literature were identical except for minor chemical shifts observed for H-3, NH and OH. (Table S2b). These data together with the observed specific rotation data $[\alpha]^{25}_D$ – 21.4 (*c* 0.2, MeOH), [lit.[24] $[\alpha]^{26}_D$ – 24.4 (*c* 0.29, EtOH)] confirmed the structure of the isolated compound as the known korormicin (**1**).

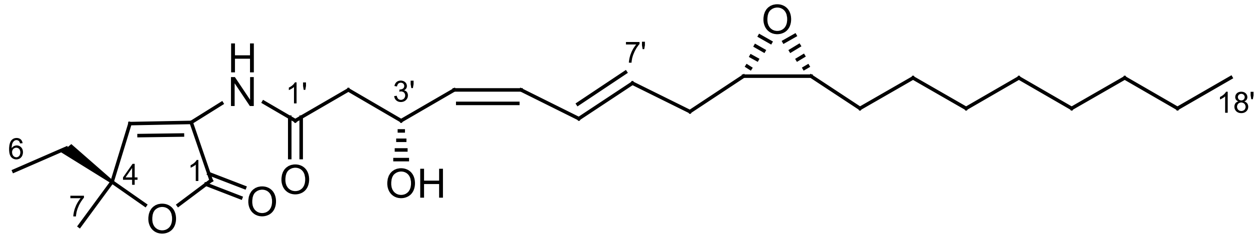


**Supplementary Figure S2.** Korormicin (**1**)

*Monitoring McH1-7 colonization over time through ddPCR*

One fragment of each genotype was sacrificed at the following time points: before inoculation with McH1-7, and 1 day, 3 days, 7 days, 21 days, and 28 days post-inoculation for a total of 24 coral samples. Coral fragments were removed from the aquaria, frozen immediately, and stored at –20 ºC until DNA extraction. Tissue and mucus were scraped from the coral fragment and DNA was extracted from the tissue/mucus slurry with a Qiagen DNeasy PowerSoil Kit. Water samples from each aquarium were collected immediately before inoculation with McH1-7, approximately 1 hour after inoculation, and at 1 day, 3 days, 7 days, 21 days, and 28 days post-inoculation for a total of 28 water samples. Water samples were stored in 15-ml sterile conical tubes at –20 ºC until DNA extraction. Water was filtered through a 0.22 µm Sterivex-GP filter and DNA was extracted from the filter with a Qiagen DNeasy PowerSoil Kit.

Primers for ddPCR were designed to target part of the Kor23 gene in the korormicin biosynthetic gene cluster, designated KOR F (5'- ACGTTACCCGCTATCTGTGG-3') and KOR R (5'- CGCTTTCCTAAAGCACTTGG-3'). Standard end-point PCR was performed in 25-µl reactions with One*Taq* 2× Master Mix (New England Biolabs) and 0.5 µM concentrations of each primer. Following an initial denaturation at 94°C for 3 min, thermocycling proceeded with 35 cycles of 94°C for 30 sec, 60°C for 30 sec, and 72°C for 30 sec, followed by a final extension of 72°C for 3 min. The 399-bp amplification product was confirmed by visualization on a 1.0%

agarose gel stained with ethidium bromide using DNA extracted from pure culture of McH1-7 as a template. Melt curve analysis was performed after quantitative PCR amplification on a StepOnePlus real-time PCR system (Applied Biosystems) with the following thermocycling conditions: initial denaturation at 95°C for 20 sec, followed by 40 cycles of 95°C for 3 sec and 60°C for 30 sec and a melt curve stage of 95°C for 15 sec, 60°C for 1 min, and 95°C for 15 sec. Melt curve analysis showed a single peak at 84.7°C. Droplet digital PCR was performed at the University of Florida Interdisciplinary Center for Biotechnology Research with an annealing temperature of 60°C and 1 µl of DNA per reaction. Triplicate ddPCR was performed for each sample.

To examine the impact of McH1-7 inoculation on the coral microbiome, the microbial community composition of the 24 tissue/mucus samples was characterized by amplifying the V4 region of the 16S rRNA gene as previously described [5] and sequencing on an Illumina MiSeq at the Interdisciplinary Center for Biotechnology Research at the University of Florida. Analysis and plotting of the ddPCR and 16S amplicon data were performed in R as described at https://github.com/meyermicrobiolab/McH1-7_Probiotics_Trials.

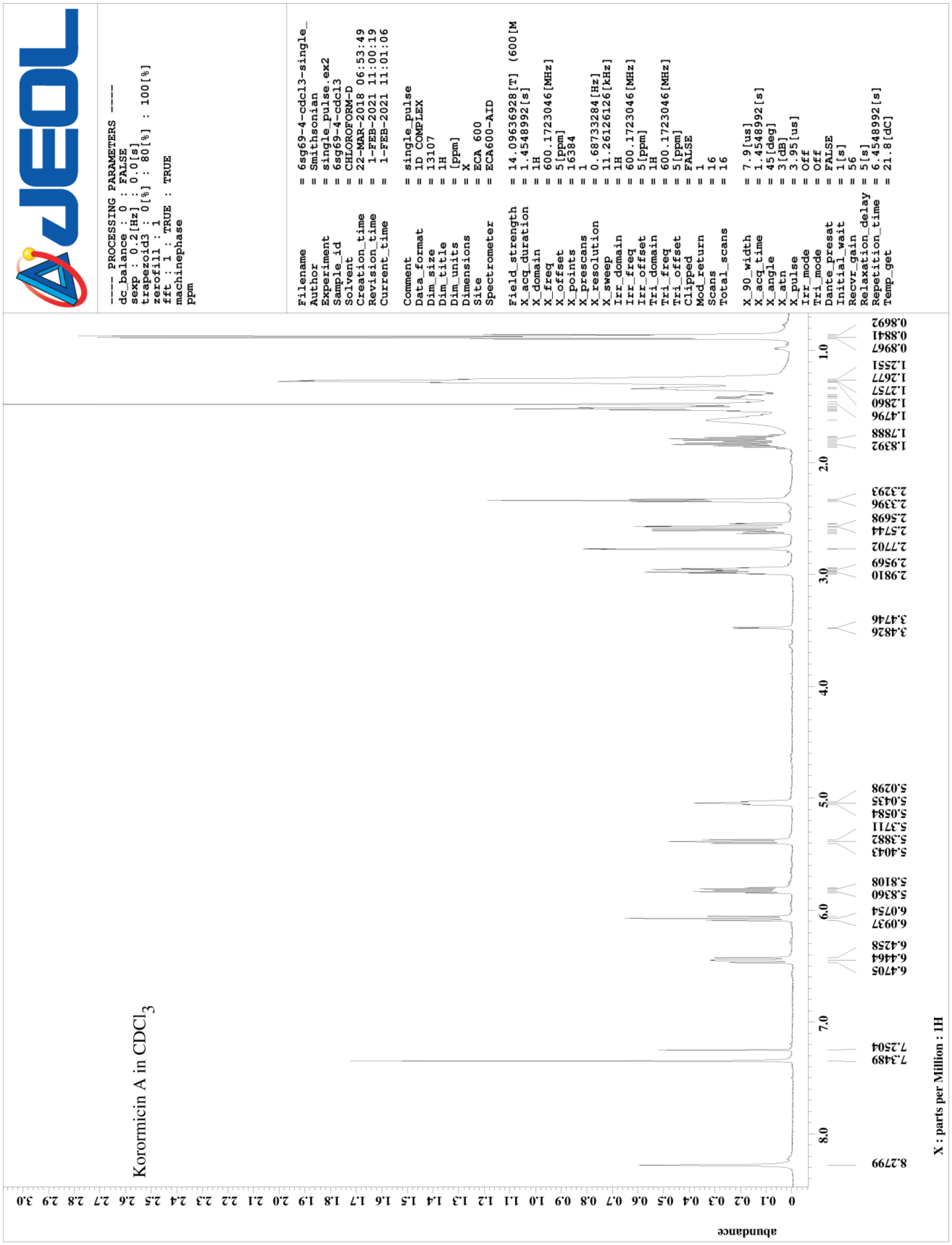


**Supplementary Figure S3.**
$^1$H NMR (600 MHz, $CDCl_3$) spectrum of korormicin A

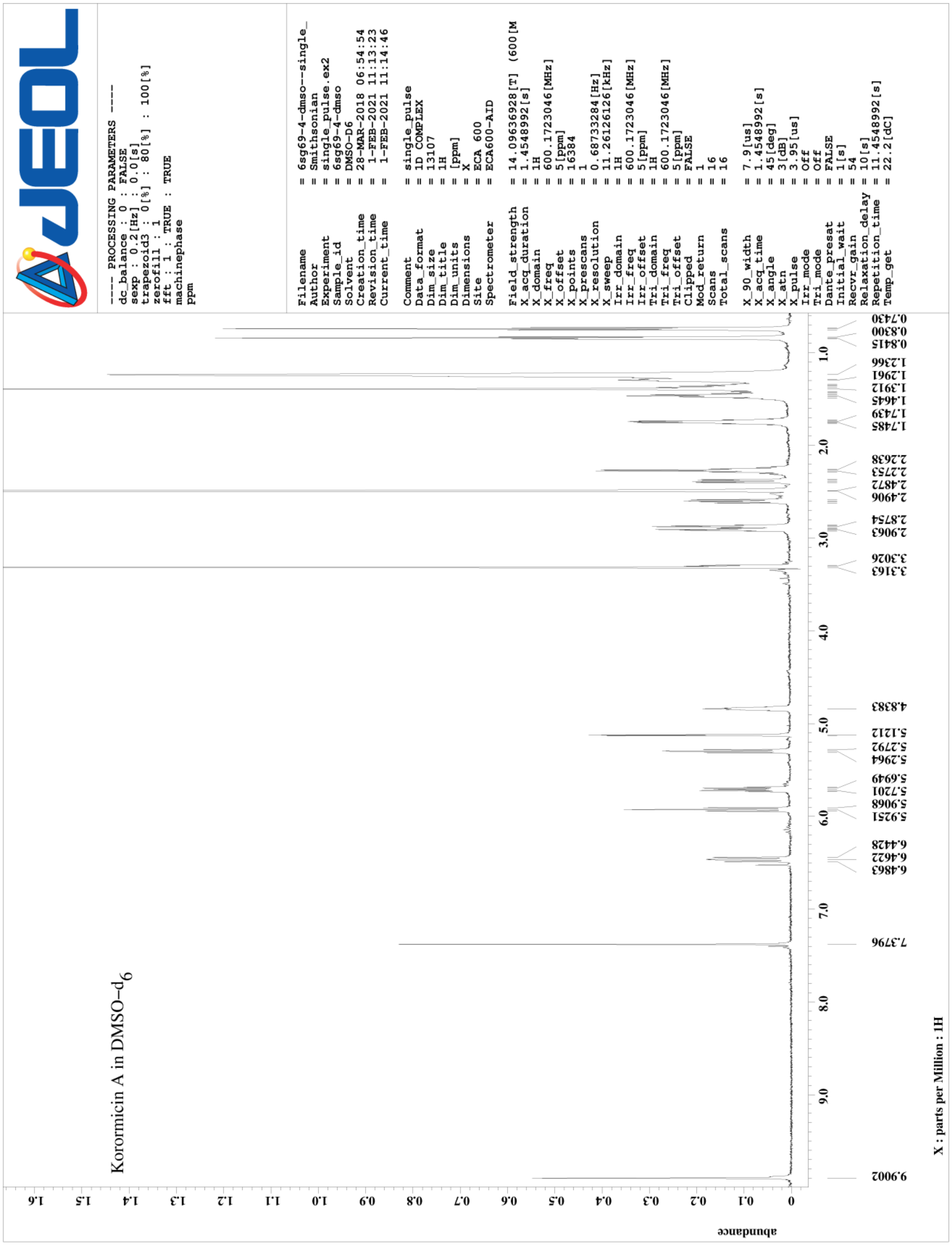


**Supplementary Figure S4.**

$^1$H NMR (600 MHz, DMSO-$d_6$) spectrum of korormicin A

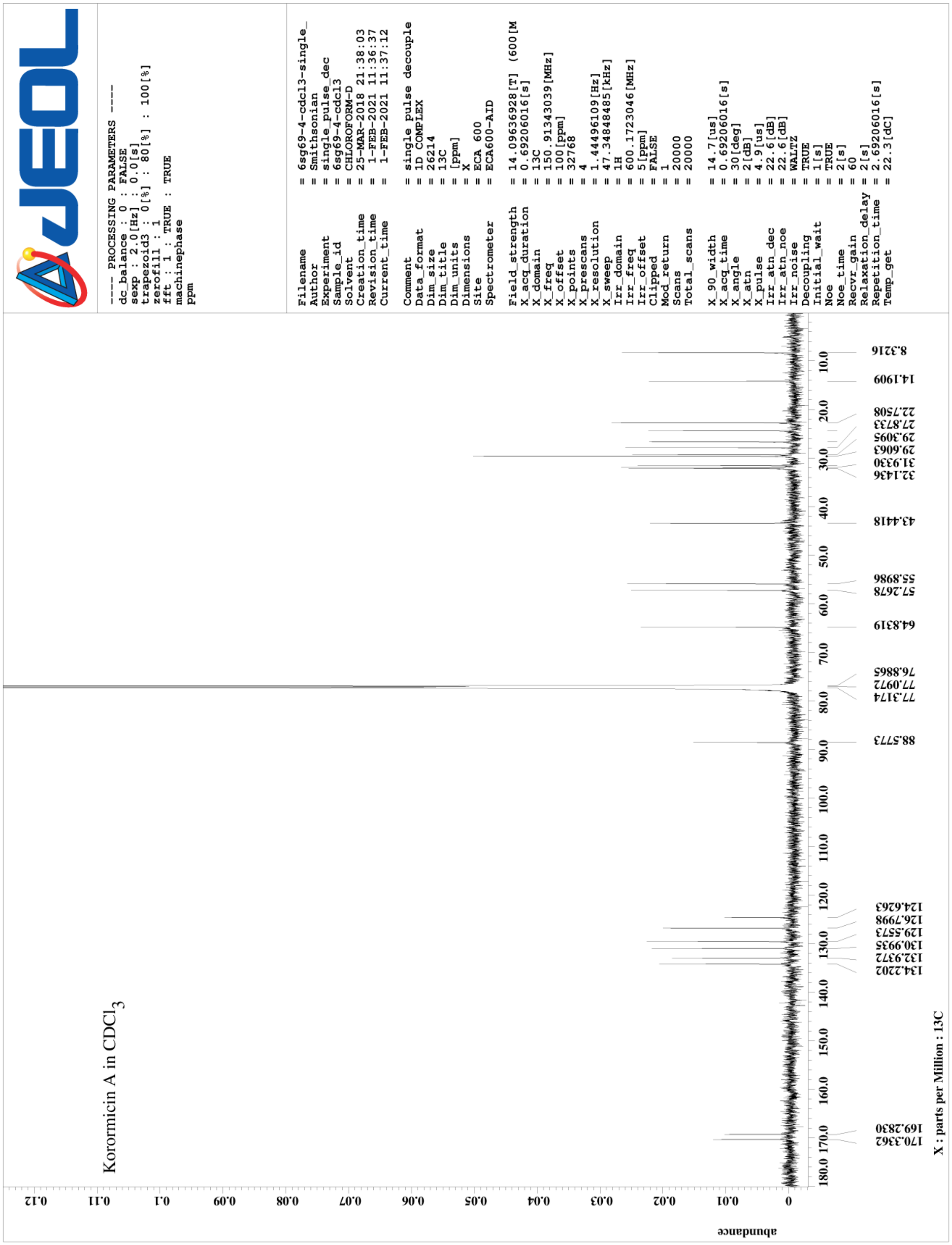

**Supplementary Figure S5.**
$^{13}C$ NMR (151 MHz, $CDCl_3$) spectrum of korormicin A

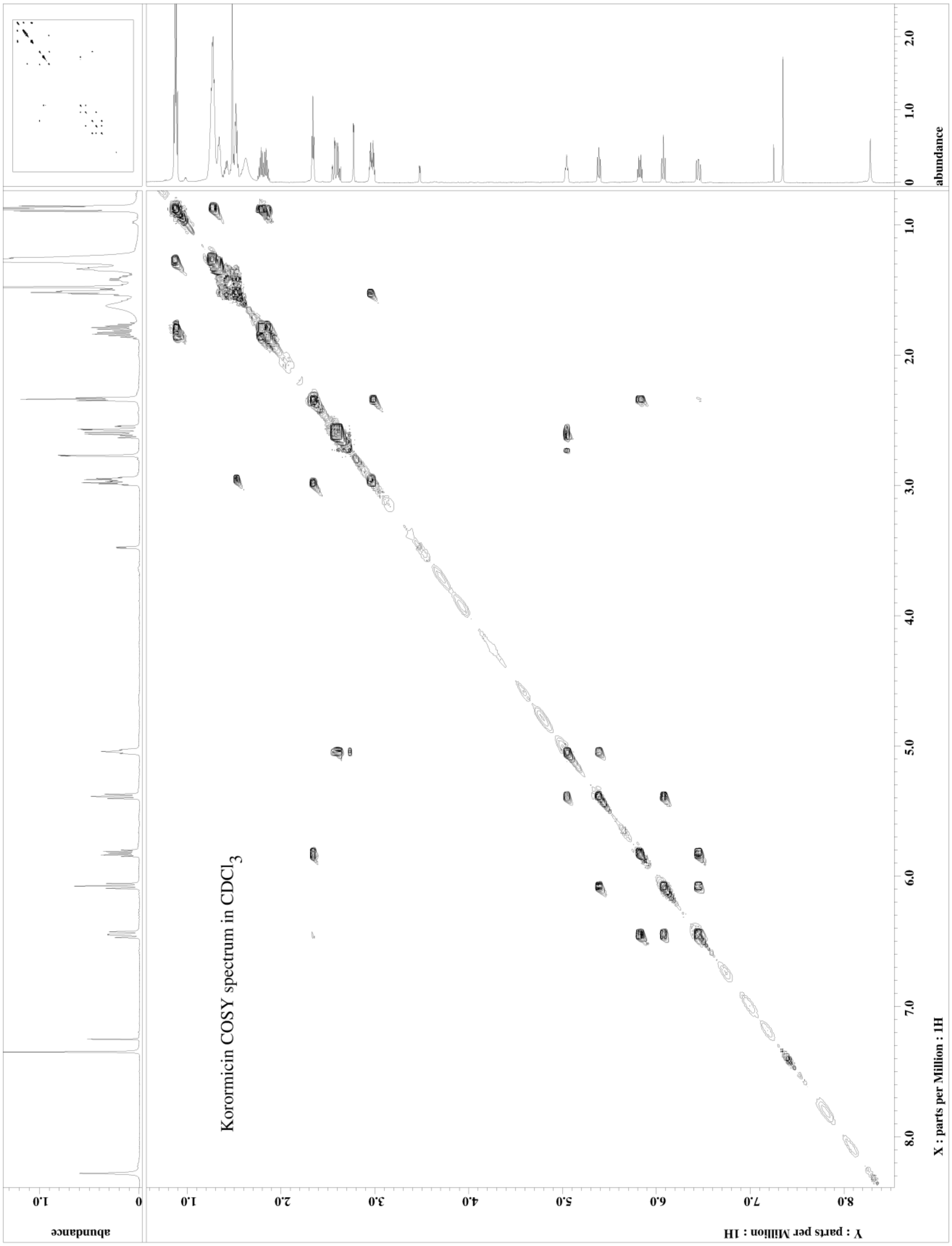


**Supplementary Figure S6.**
DQF-COSY NMR (600 MHz, $CDCl_3$) spectrum of korormicin A

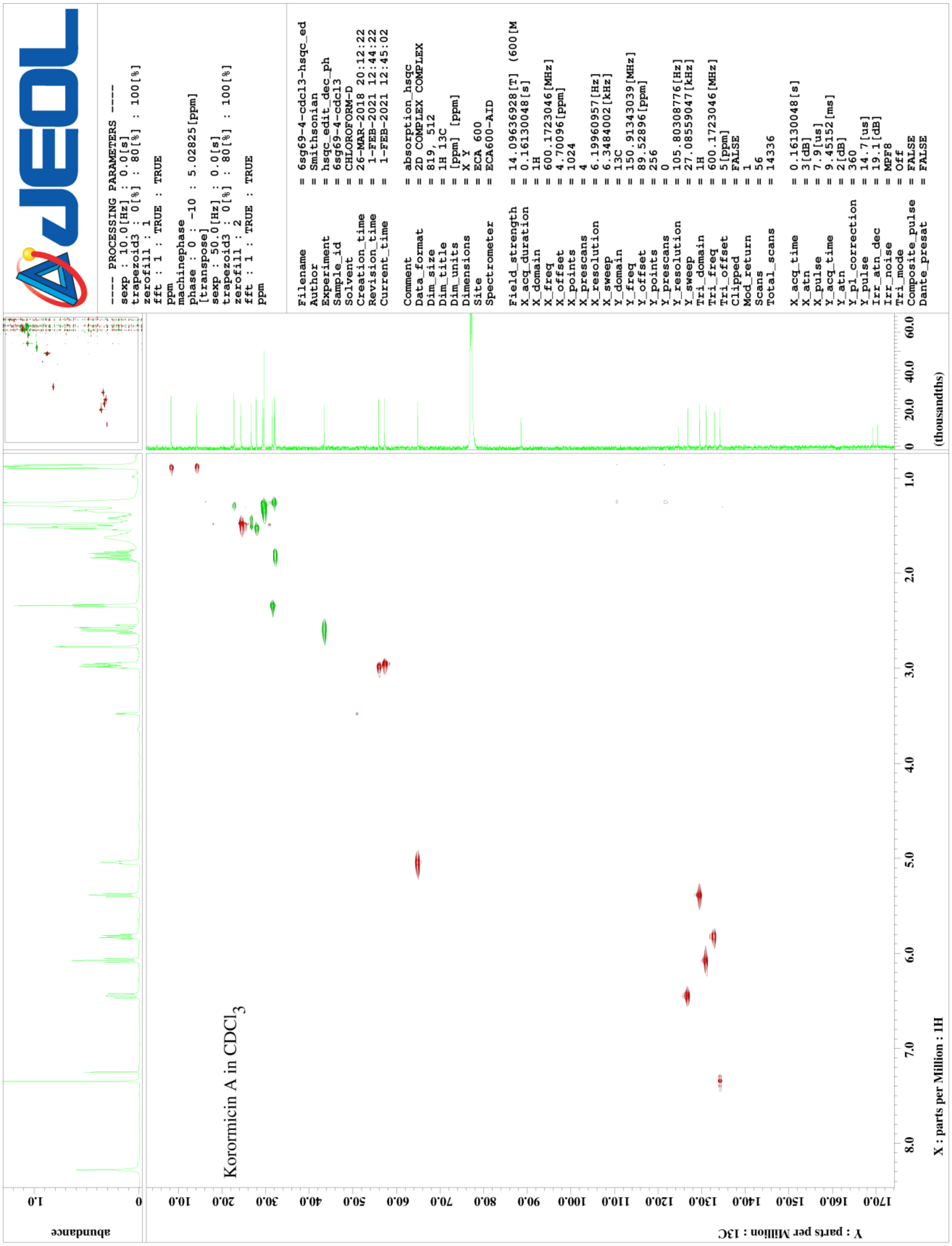


**Supplementary Figure S7.**
HSQC NMR (600 MHz, $CDCl_3$) spectrum of korormicin A

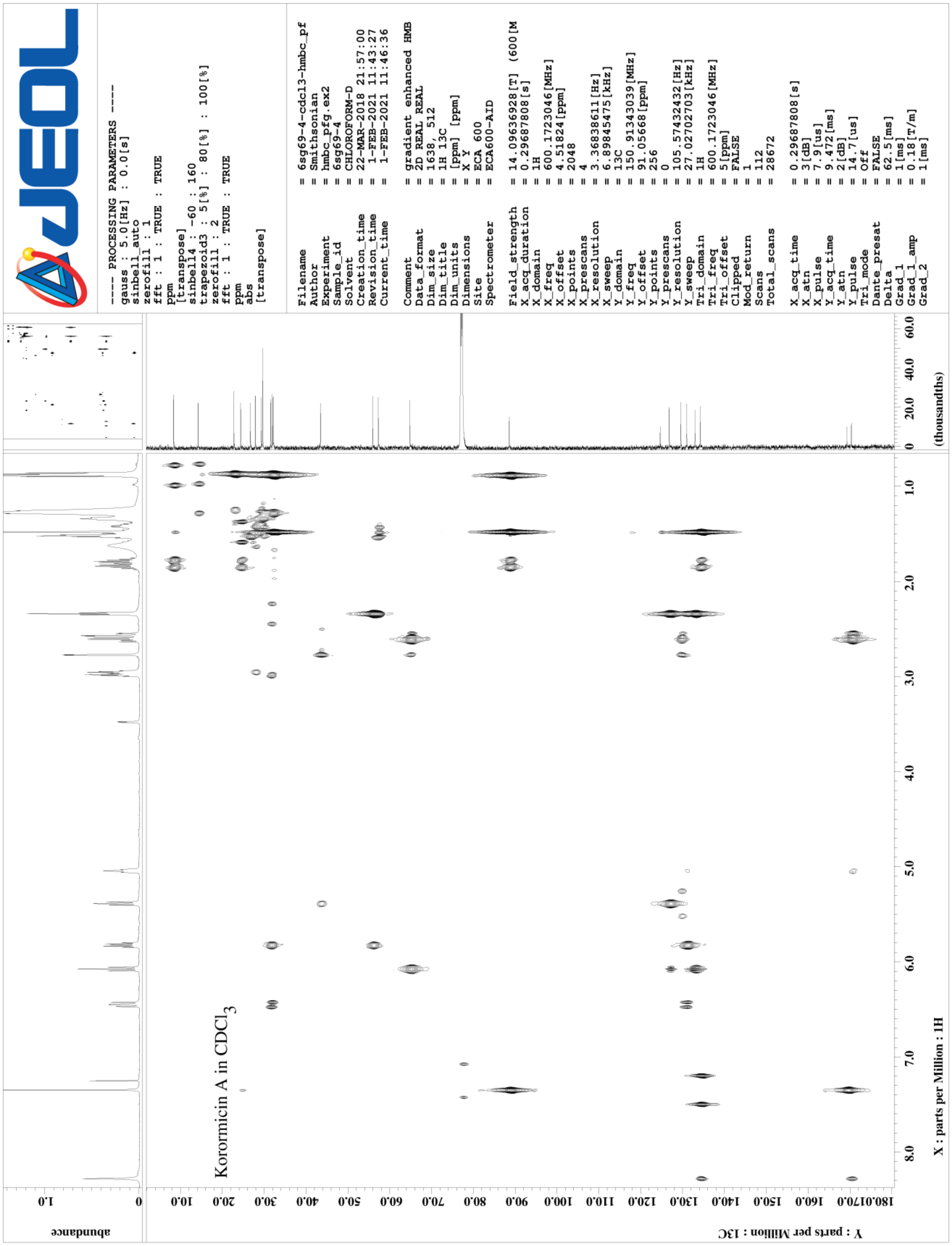


**Supplementary Figure S8.**
HMBC NMR (600 MHz, $CDCl_3$) spectrum of korormicin A

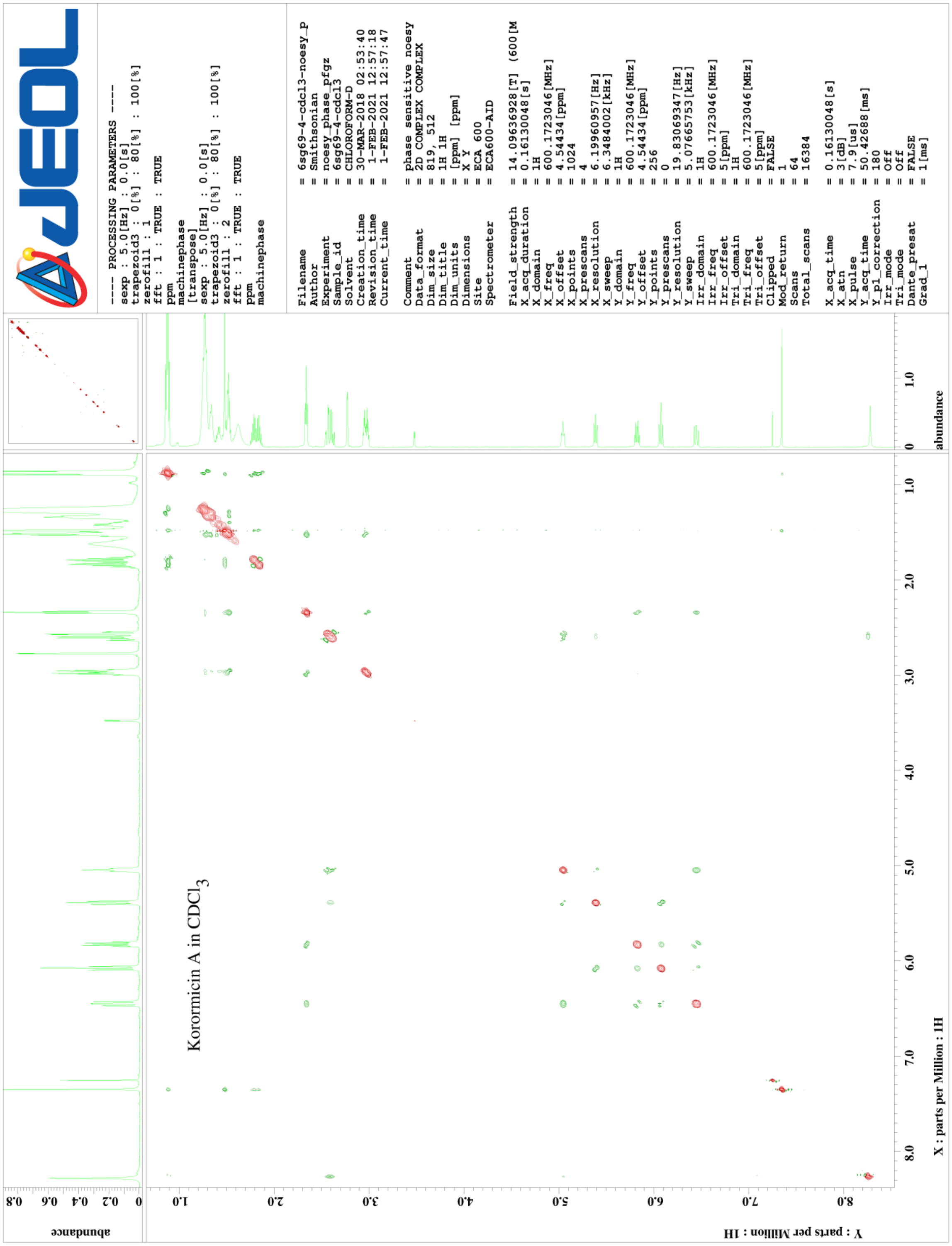


**Supplementary Figure S9.**
2D-NOESY NMR (600 MHz, $CDCl_3$) spectrum of korormicin A

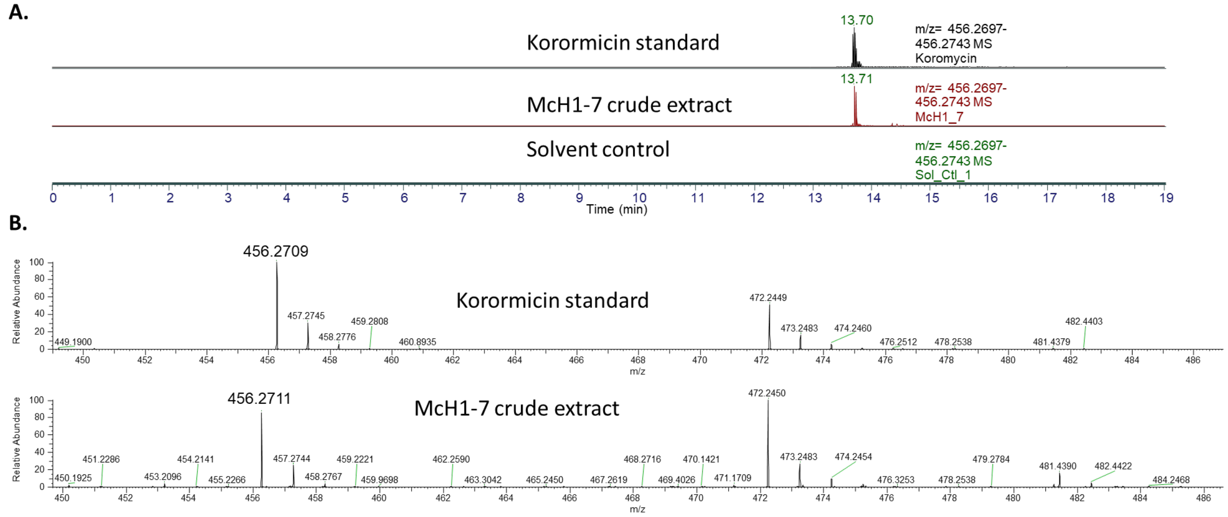


**Supplementary Figure S10.**
Korormicin A was produced by McH1-7 in the LC-HRMS analysis. A) Extracted ion chromatograms (EIC) of standard korormicin A, crude extract of McH1-7, and solvent control. B) HRMS spectra of the peak content with a retention time of 13.7 min in the EIC traces of standard korormicin A (top) and crude extract of McH1-7 (bottom). Of note, the $[M+K]^+$ ion of korormicin A was also observed in both traces and identical (472.2449 vs 472.2450).

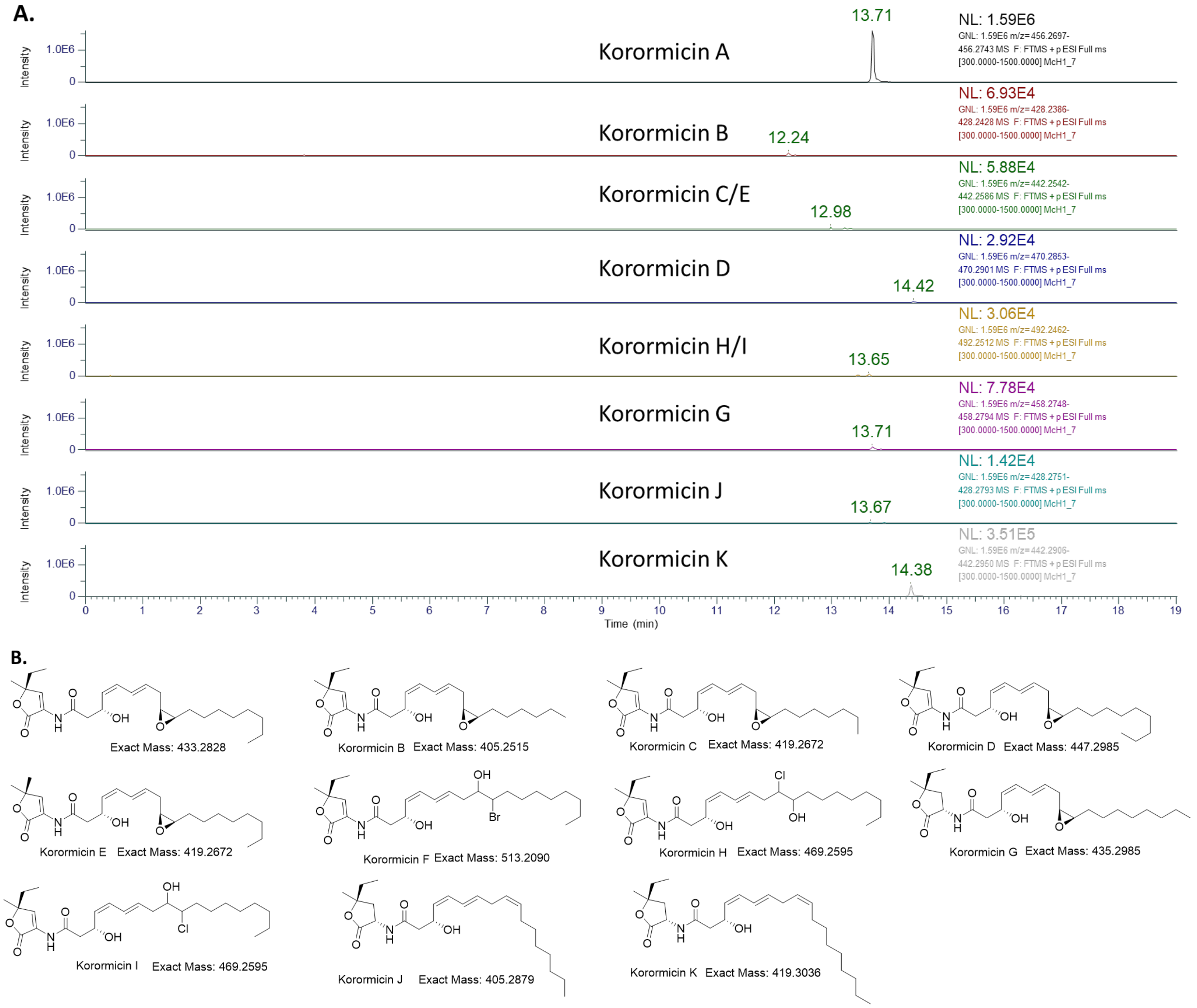


**Supplementary Figure S11.**
Multiple putative korormicin analogs were detected in the crude extract of McH1-7 in the LC-HRMS analysis. A) EIC traces of korormicin A and its analogs. The intensities of all other analogs were 5 to 100 times lower than korormicin A. Korormicin F was not found here. The exact masses of korormicin C and E are the same and are not able to be differentiated in the EIC analysis, and the same for korormicin H and I. B) Chemical structures and extract masses of known korormicin analogs.

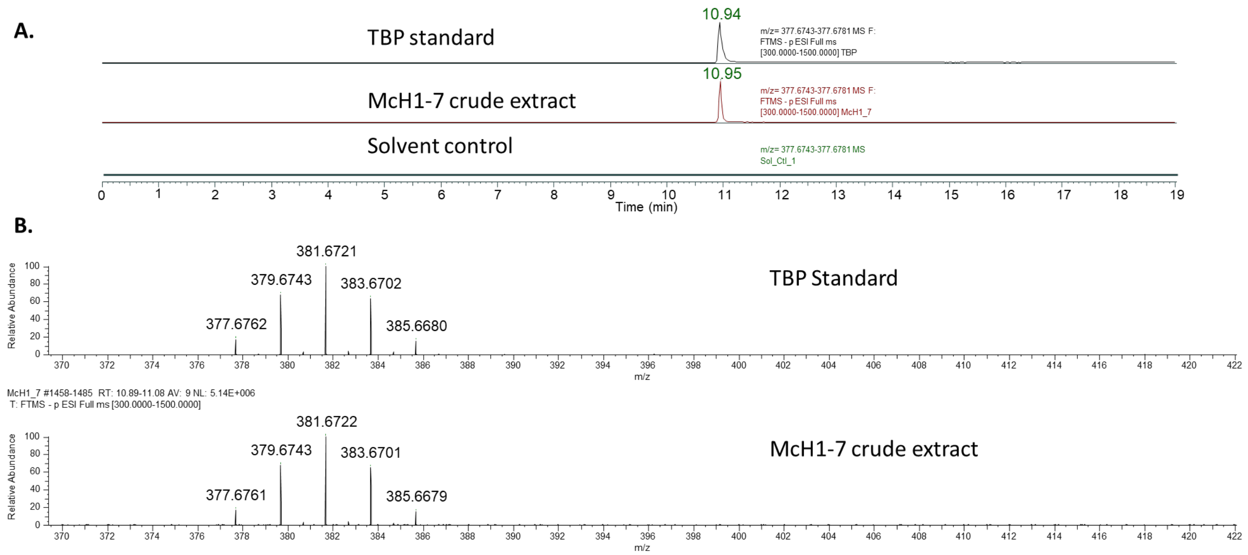


**Supplementary Figure S12.**

TBP was produced by McH1-7 in the LC-HRMS analysis and detected with the negative ion mode. A) Extracted ion chromatograms (EIC) of standard TBP, crude extract of McH1-7, and solvent control. B) MS spectra of the peak content with a retention time of 10.9 min in the EIC traces of standard TBP (top) and crude extract of McH1-7 (bottom).

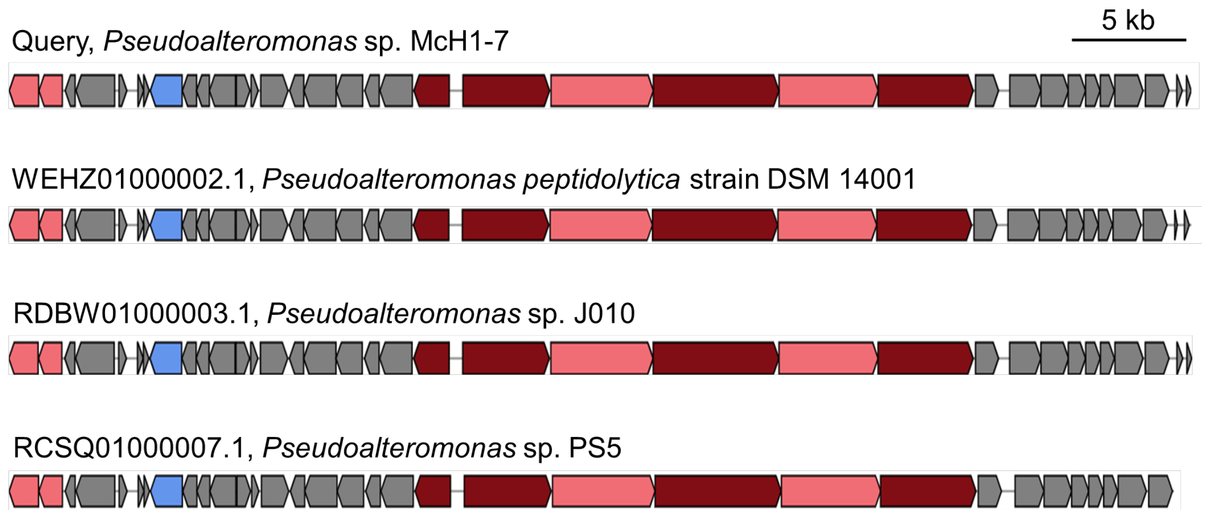


**Supplementary Figure S13.**
The distribution of the korormicin gene clusters in genomes of multiple *Pseudoalteromonas* strains. The 52-kb *kor* gene cluster from the genome of *Pseudoalteromonas* McH1-7 was used to mine the genomes available in NCBI GenBank. Three almost identical gene clusters were identified from *Pseudoalteromonas peptidolytica* DSM 14001, *Pseudoalteromonas* sp. J010 and PS5.

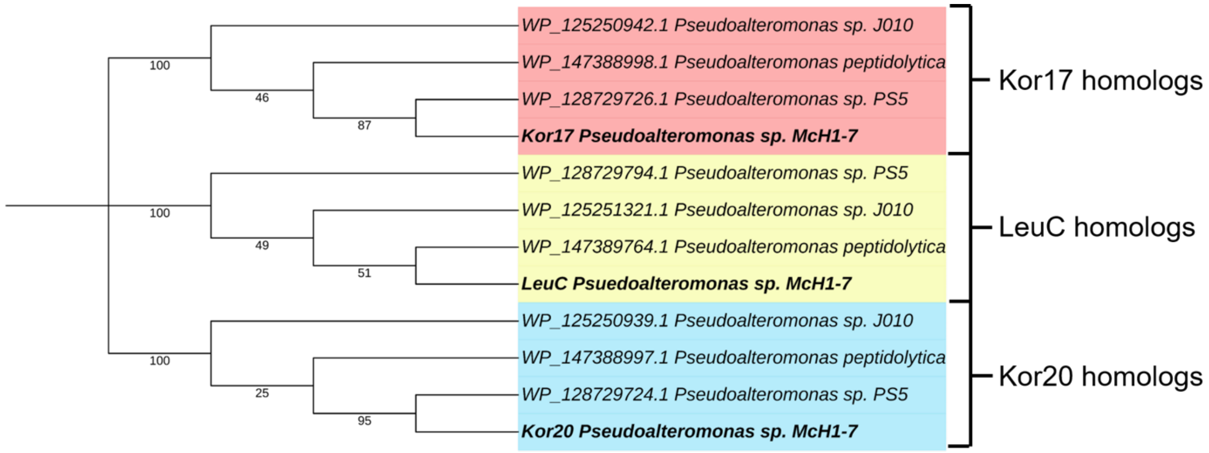


**Supplementary Figure S14.**
Phylogenetic tree of the two 3-isopropylmalate dehydratase large subunits within the *kor* gene cluster. Phylogenetic analysis reveals that the two 3-isopropylmalate dehydratase large subunits within the *kor* gene cluster are different with each other and the housekeeping enzyme LeuC. The phylogenetic tree was generated with MEGAX. The enzyme homologs encoded by the *kor* gene clusters (Kor17 and Kor20) and the housekeeping enzymes (LeuC) form different clades.

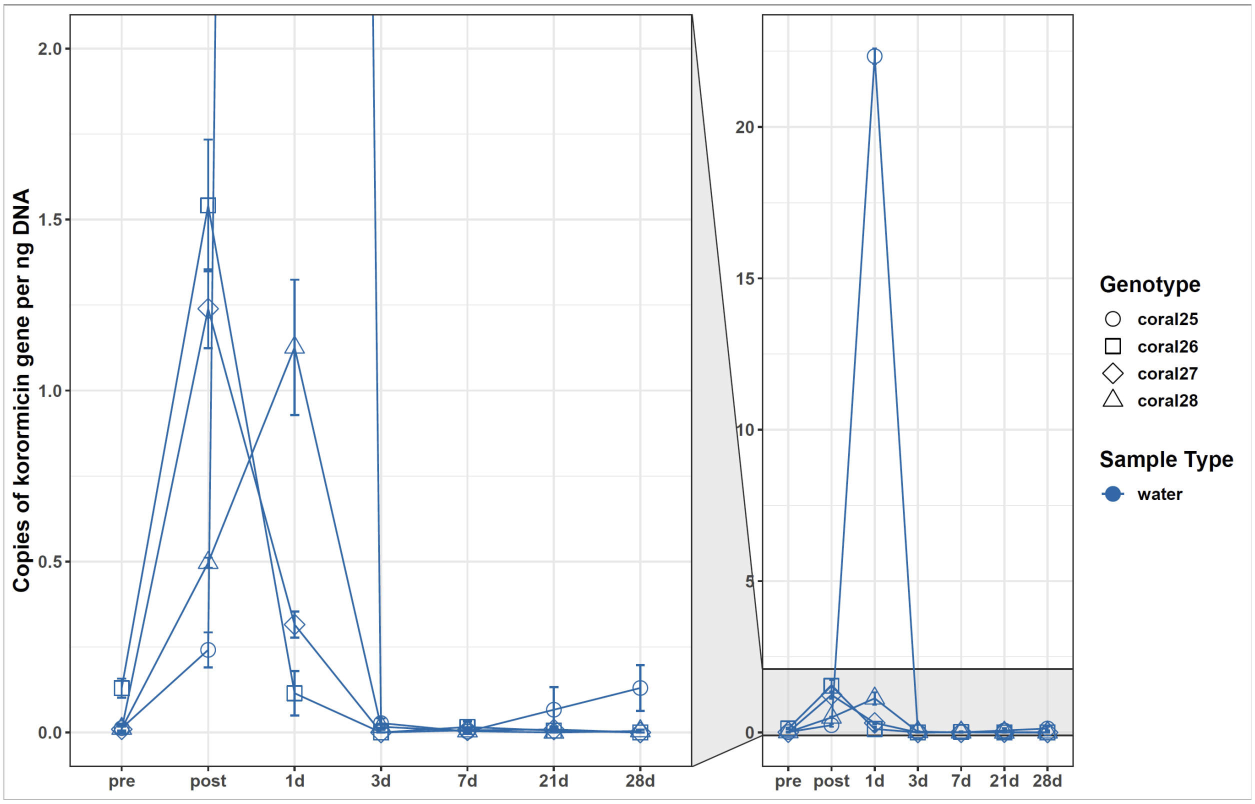


**Supplementary Figure S15.**
Detection of korormicin Kor 23 gene copies over time after inoculation with *Pseudoalteromonas* sp. McH1-7. The full data range (0 to 25 gene copies per ng DNA) is shown on the right and on the left, the graph is zoomed into 0 to 2 gene copies per ng DNA. Points show the mean copy number per ng of DNA from triplicate reactions of ddPCR and the standard error is shown by the error bars. "Pre" indicates samples taken before inoculation with the probiotic strain McH1-7. "Post" indicates samples taken one hour after inoculation in water samples only.

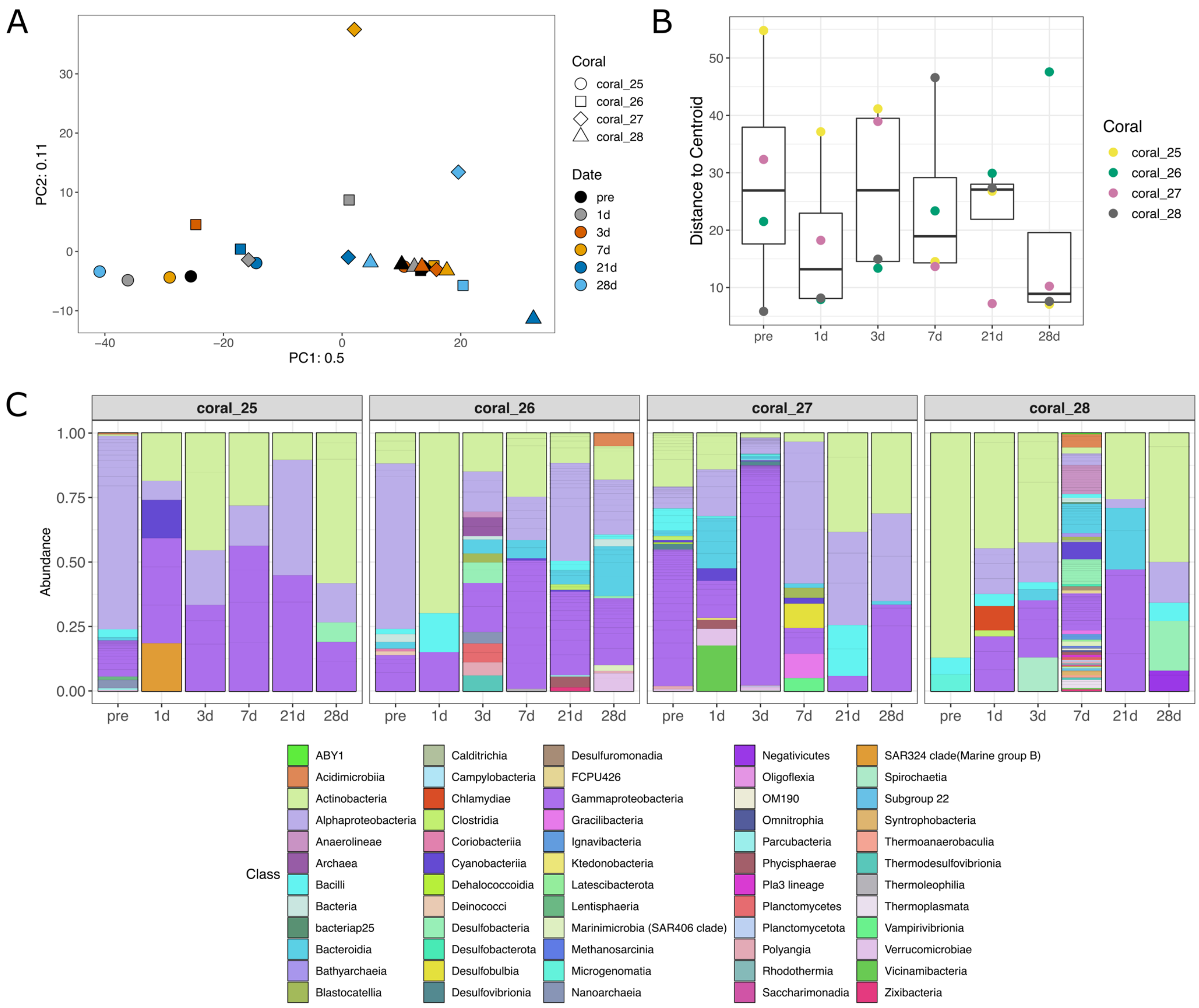


**Supplementary Figure S16.**

**Microbial community composition of healthy corals before and after inoculation with *Pseudoalteromonas* sp. McH1-7.** A) Principal components analysis of the Aitchison distance among tissue/mucus samples from four coral genotypes over six timepoints (before inoculation and 1 – 28 days post inoculation). B) Distance to Centroid from four coral genotypes over six timepoints. C) Relative abundance of amplicon sequence variants colored by class in the same 24 tissue/mucus samples as in panel A. "Pre" indicates samples taken before inoculation with *Pseudoalteromonas* sp. McH1-7.

**Supplementary Table S1.**
CFU calculations and inhibitory isolate counts from *M. cavernosa* fragments exposed to SCTLD.

| Coral ID | Transmission outcome | Inhibitory isolates (inhibitory/total) | Mean SWA CFU $ml^{-1}$ | Mean TCBS agar CFU $ml^{-1}$ |
|---|---|---|---|---|
| **McH1** | Healthy | 67/111 | $2.37 \times 10^5$ | $1.03 \times 10^4$ |
| **McH2** | Diseased | 1/111 | $4.60 \times 10^5$ | $1.02 \times 10^5$ |
| **McH3** | Diseased | 0/111 | $1.12 \times 10^5$ | $1.60 \times 10^3$ |
| **McH4** | Healthy | 16/111 | $6.17 \times 10^5$ | $3.51 \times 10^4$ |

**Supplementary Table S2**.
NMR Spectroscopic Data for korormicin A in $CDCl_3$ (600 MHz)

| position | $\delta_C$ mult. | $\delta_H$ ($J$ in Hz) | COSY[a] | HMBC | NOESY[b] |
|---|---|---|---|---|---|
| 1 | 169.2, C | | | 3 | |
| 2 | 126.67, C | | | | |
| 2 NH | | 8.27, s | | | 2'a, 2'b |
| 3 | 134.1, CH | 7.34, s | | 5, 7, NH | 7 |
| 4 | 88.4, C | | | 6, 7 | |
| 5 | 32.0, $CH_2$ | 1.83, m | 6 | 7 | 6 |
| | | 1.78, m | 6 | | 6 |
| 6 | 8.2, $CH_3$ | 0.88, t (6.9) | 5 | 5 | 5 |
| 7 | 24.2, $CH_3$ | 1.47, s | | 3, 5 | 3, 5 |
| 1' | 170.2, C | | | NH, 2', 3' | |
| 2a' | 43.3 $CH_2$ | 2.60, dd (15.1, 8.1) | 3' | 3', 4' | NH, 4' |
| 2b' | | 2.56, dd (15.1, 3.4) | 3' | | NH, 4' |
| 3' | 64.7, CH | 5.04, m | 2'-ab, 3'-OH, 4' | 2', 4', 3'-OH | 4', 6' |
| 3' OH | | 2.77, d (2.8) | 3' | 2', 3' | |
| 4' | 129.4, CH | 5.38, dd (10.9, 9.0) | 3', 5' | 2', 6' | 2', 3', 5' |
| 5' | 130.8, CH | 6.09, dd (11.2, 10.9) | 4', 6' | 3', 4', 6', 7' | 4', 6', 7' |
| 6' | 126.7, CH | 6.44, dd (15.1, 11.2) | 5', 7' | 4', 5', 7' | 3', 5', 8' |
| 7' | 132.8, CH | 5.83, ddd (15.1, 6.8, 6.8) | 6', 8' | 5', 6', 8', 9' | 5', 8' |
| 8' | 31.4, $CH_2$ | 2.33, dd (6.8, 5.9) | 7', 9' | 6', 7', 9' | 7', 9', 11' |
| 9' | 55.8, CH | 2.98, dd (5.9, 4.2) | 8', 10' | 7', 8' | 8' |
| 10' | 57.1, CH | 2.95, ddd (6.1, 4.2, 4.2) | 9', 11' | 11', 12' | 8', 12' |
| 11' | 26.5, $CH_2$ | 1.53, m, 1.51, m | 10', 12' | 9', 12' | 8' |
| 12' | 27.7, $CH_2$ | 1.52, m | 11', 13' | 10' | |
| 13' | 29.2, $CH_2$ | 1.26, m | 12', 14' | | |
| 14' | 29.5, $CH_2$ | 1.26, m | | | |
| 15' | 29.5, $CH_2$ | 1.26, m | | | |
| 16' | 31.8, $CH_2$ | 1.27, m | 17' | | |
| 17' | 22.6, $CH_2$ | 1.30, m | 16', 18' | | 18' |
| 18' | 14.1, $CH_3$ | 0.86, t (7.3) | 17' | 16', 17' | 17' |

[a]COSY and NOESY correlations are from proton(s) stated to the indicated protons.
[b]*HMBC* correlations are from proton(s) stated to the indicated carbons.

**Supplementary Table S3.**
Proton NMR data comparison in DMSO-$d_6$

**Comparison of Proton NMR Spectroscopic Data of Korormicin (1) with the Published Data[a] in DMSO-$d_6$**

| position | $\delta_H$ (*J* in Hz) | $\delta_H$ (*J* in Hz)[a] |
|---|---|---|
| 2 NH | 9.90, s | 9.83, s |
| 3 CH | 7.38, s | 7.26, s |
| 5 $CH_2$ | 1.74, q (7.3) | 1.74, q (7.3) |
| 6 $CH_3$ | 0.74, t (6.9) | 0.74, t (6.9) |
| 7 $CH_3$ | 1.37, s | 1.37, s |
| 2a' $CH_2$ | 2.59, dd (8.1, 14.4) | 2.59, dd (8.1, 14.4) |
| 2b' | 2.39, dd (5.4, 14.4) | 2.39, dd (5.4, 14.4) |
| 3' CH | 4.83, m | 4.83, m |
| 3' OH | 5.12, d (4.8) | 5.09, d (4.4) |
| 4' CH | 5.30, dd (10.9, 9.0) | 5.30, dd (10.9, 9.0) |
| 5' CH | 5.92, dd (10.9, 11.2) | 5.92, dd (10.9, 11.2) |
| 6' CH | 6.46, dd (11.2, 15.1) | 6.46, dd (11.2, 15.1) |
| 7' CH | 5.70, dt (6.8, 15.1) | 5.70, dt (6.8, 15.1) |
| 8' CH | 2.26, dd (5.9, 6.8) | 2.26, dd (5.9, 6.8) |
| 9' CH | 2.90, dd (4.2, 5.9) | 2.90, dd (4.2, 5.9) |
| 10' CH | 2.87, dt (4.2, 6.1) | 2.87, dt (4.2, 6.1) |
| 11' $CH_2$ | 1.48, m | 1.48, m |
| 12' $CH_2$ | 1.38, m | 1.38, m |
| 13' $CH_2$ | 1.2-1.4, m | 1.2-1.4, m |
| 14' $CH_2$ | 1.2-1.4, m | 1.2-1.4, m |
| 15' $CH_2$ | 1.2-1.4, m | 1.2-1.4, m |
| 16' $CH_2$ | 1.22, m | 1.22, m |
| 17' $CH_2$ | 1.24, m | 1.24, m |
| 18' $CH_3$ | 0.83, t (7.1) | 0.83, t (7.1) |

[a]Yoshikawa, K., Takadera, T., Adachi, K., Nishijima, M. & Sanc, H. Korormicin, a novel antibiotic specifically active against marine Gram-negative bacteria, produced by a marine bacterium. *The Journal of Antibiotics* **50**, 949–953 (1997).

**Table S4.**
*M. cavernosa* colonies used McH1-7 direct treatment experiments. BC = Broward Country; Keys = FL Keys.

| Colony ID | Collection site | Collection date | VcpA test? | Control | McH1-7 treatment |
|---|---|---|---|---|---|
| 9 | BC | 10/18/2019 | Not done | Slow | Stop- day 17 |
| D | BC | 10/18/2019 | Not done | Slow | Stop- day 3 |
| 18 | BC | 10/18/2019 | Not done | Slow | Slow |
| C | BC | 10/18/2019 | Not done | Dead- day 11 | Stop- day 17 |
| McD-11 FtL | BC | 5/9/2019 | - | Dead- day 8 | Stop- day 7 |
| McD-12 FtL | BC | 5/9/2019 | - | Slow | Stop- day 13 |
| McD-14 FtL | BC | 5/9/2019 | - | Slow | Stop- day 13 |
| McD-15 FtL | BC | 5/9/2019 | - | Slow | Stop- day 13 |
| McD-16 FtL | BC | 5/9/2019 | + | Dead- day 9 | Stop- day 13 |
| McD-17 FtL | BC | 5/9/2019 | + | Dead- day 2 | Dead- day 8 |
| McD-19 FtL | BC | 6/28/2022 | - | Dead- day 6 | Stop- day 15 |
| McD-2 | Keys | 1/30/2019 | - | Slow | Stop- day 7 |
| McD-4 | Keys | 1/30/2019 | - | Dead- day 8 | Dead- day 15 |
| McD-7 | Keys | 1/30/2019 | + | Dead- day 2 | Dead- day 4 |
| McD-8 | Keys | 1/30/2019 | + | Dead- day 3 | Dead- day 7 |
| McD-21 | Keys | 5/3/2019 | + | Dead- day 3 | Dead- day 7 |
| McD-33 | Keys | 10/11/2019 | + | Dead- day 12 | Stop- day 13 |
| McD-35 | Keys | 10/11/2019 | - | Slow | Dead- day 7 |
| McD-36 | Keys | 10/11/2019 | + | Dead- day 21 | Slow |
| McD-49 | Keys | 6/12/2020 | - | Dead- day 12 | Dead- day 13 |
| McD-51 | Keys | 6/12/2020 | - | Dead- day 20 | Stop- day 15 |
| McD-53 | Keys | 6/12/2020 | + | Slow | Stop- day 17 |

**Table S5.**
*M. cavernosa* colonies used in McH1-7 prophylactic experiments. BC = Broward Country; Keys = FL Keys; Con tank = diseased fragment in contact with non-treated healthy fragment; Con transmit = was there disease transmission to the non-treated fragment; Exp tank = diseased fragment in contact with treated healthy fragment; Exp transmit = was there disease transmission to the treated fragment.

| Disease ID | Collect site | Collect Date | VcpA test? | Con tank | Con transmit? | Exp tank | Exp transmit? | Healthy colony ID | Healthy collection |
|---|---|---|---|---|---|---|---|---|---|
| McD-4 FtL | BC | 3/26/2019 | - | Slow | No | Stop- day 5 | No | McH-A | Key West Nursery |
| McD-5 FtL | BC | 3/26/2019 | - | Dead- day 2 | No | Stop- day 5 | No | McD-B | Key West Nursery |
| McD-8 FtL | BC | 3/26/2019 | - | Dead- day 14 | No | Slow | No | McH-C | Key West Nursery |
| McD-9 FtL | BC | 3/26/2019 | - | Dead- day 17 | No | Slow | No | McH-D | Key West Nursery |
| McD-18 FtL | BC | 6/28/2019 | - | Dead- day 7 | Yes- day 6 | Dead- day 10 | No | McH-A2 | Key West Nursery |
| McD-19 FtL | BC | 6/28/2019 | - | Slow | No | Slow | No | McH-B2 | Key West Nursery |
| McD-21 FtL | BC | 6/28/2019 | - | Slow | No | Slow | No | Mc-10 | Key West Nursery |
| McD-22 FtL | BC | 6/28/2019 | + | Dead- day 6 | Yes- day 5 | Dead- day 20 | No | Mc-8 | Key West Nursery |
| McD-12 | Keys | 4/4/2019 | - | Dead- day 7 | Yes- day 5 | Slow | No | McH-E | Key West Nursery |
| McD-13 | Keys | 4/4/2019 | - | Slow | No | Stop- day 3 | No | McH-F | Key West Nursery |
| McD-14 | Keys | 4/4/2019 | - | Dead- day 5 | Yes- day 4 | Slow | No | McH-G | Key West Nursery |
| McD-17 | Keys | 4/4/2019 | - | Dead- day 3 | No | Dead- day 11 | No | McH-J | Key West Nursery |

**Table S6.**
List of all bacterial strains and plasmids used in this study.

| Bacterial isolates | Description | Citation/Source |
|---|---|---|
| *Pseudoalteromonas* sp. McH1-7 | Marine isolate with antibacterial activity; a disease-resistant *M. cavernosa;* FL, USA. | [7] |
| *Alteromonas* sp. McT4-15 | Coral isolate; SCTLD lesion; FL, USA. | [7] |
| *Alteromonas* sp. MmMcT2-2 | Coral isolate; SCTLD lesion; FL, USA. | [7] |
| *Alteromonas* sp. CNT1-3 | Coral isolate; SCTLD lesion; FL, USA. | [7] |
| *Leisingera* sp. McT4-56 | Coral isolate; SCTLD lesion; FL, USA. | [7] |
| *Thalassobius* sp. CNT1-3 | Coral isolate; SCTLD lesion; FL, USA. | [7] |
| *V. coralliilyticus* OfT6-21 | Coral isolate; SCTLD lesion; FL, USA. | [7] |
| *V. coralliilyticus* OfT7-21 | Coral isolate; SCTLD lesion; FL, USA. | [7] |
| *V. coralliilyticus* MmMcT2-4 | Coral isolate; SCTLD lesion; FL, USA. | [7] |
| *V. coralliilyticus* ATCC BAA-450 | Coral pathogen; coral isolate; Zanzibar | ATCC |
| *V. coralliilyticus* OCN008 | Coral pathogen; coral isolate; HI, USA | [7] |
| *V. coralliilyticus* OCN014 | Coral pathogen; coral isolate; Palmyra Atoll, Line Islands | [7] |
| *V. coralliilyticus* RE22 | Shellfish pathogen; oyster larvae; OR, USA. | [7] |
| *V. coralliilyticus* RE98 | Shellfish pathogen; oyster larvae; OR, USA. | [7] |
| *V. owensii* OCN002 | Coral pathogen; coral isolate; HI, USA | Laboratory Archive |
| *V. tubiashii* ATCC 19109 | Shellfish pathogen; clam larvae; CT, USA. | ATCC |
| *V. tasmaniensis* LGP32 | Shellfish pathogen; adult oyster; La Tremblade, France. | [70] |
| *V. harveyi* ATCC BAA-1118 | Marine bacterium; NJ, USA. | ATCC |
| *V. fluvialis* 1958-82 | Human pathogen; clinical isolate; HI, USA | Laboratory Archive |
| *V. alginolyticus* ATCC 17749 | Human pathogen; clinical isolate; China. | ATCC |
| *V. mimicus* ATCC 33653 | Human pathogen; clinical isolate; NC, USA. | ATCC |
| *V. parahaemolyticus* ATCC 17802 | Human pathogen; clinical isolate; Japan. | ATCC |
| *V. vulnificus* ATCC 27562 | Human pathogen; clinical isolate; FL, USA. | ATCC |
| *V. cholerae* O395N1 (classical) | Human pathogen; clinical isolate; India. | Laboratory Archive |
| *V. cholerae* N16961 (El Tor) | Human pathogen; clinical isolate; Bangladesh. | Laboratory Archive |
| *Pseudoalteromonas piratica* OCN003 | Coral pathogen; coral isolate; HI, USA. | Laboratory Archive |
| *E. coli* DH5α | Laboratory strain; Gram-negative isolate. | Laboratory Archive |
| *Pseudomonas aeruginosa* PAO1 | Human pathogen; clinical isolate; Australia. | Laboratory Archive |
| *Serratia marcescens* 155450A | Non-pathogenic; Gram-negative isolate. | Carolina Biological |
| *Proteus vulgaris* 155240A | Human pathogen; Gram-negative isolate. | Carolina Biological |
| *Staphylococcus aureus* 155554A | Human pathogen; Gram-positive isolate. | Carolina Biological |
| *Bacillus subtilis* 154921A | Non-pathogenic; Gram-positive isolate. | Carolina Biological |

**Table S7.**
Summary of Clusters of Orthologous Gene (COG) categories in the genome of *Pseudoalteromonas* sp. McH1-7, based on the IMG Annotation Pipeline.

| COG category | Count | Percent |
|---|---|---|
| Amino acid transport and metabolism | 303 | 7.77 |
| Carbohydrate transport and metabolism | 134 | 3.44 |
| Cell cycle control, cell division, chromosome partitioning | 45 | 1.15 |
| Cell motility | 142 | 3.64 |
| Cell wall/membrane/envelope biogenesis | 249 | 6.39 |
| Chromatin structure and dynamics | 3 | 0.08 |
| Coenzyme transport and metabolism | 162 | 4.16 |
| Cytoskeleton | 2 | 0.05 |
| Defense mechanisms | 130 | 3.34 |
| Energy production and conversion | 187 | 4.8 |
| Extracellular structures | 42 | 1.08 |
| Function unknown | 249 | 6.39 |
| General function prediction only | 324 | 8.31 |
| Inorganic ion transport and metabolism | 196 | 5.03 |
| Intracellular trafficking, secretion, and vesicular transport | 93 | 2.39 |
| Lipid transport and metabolism | 157 | 4.03 |
| Mobilome: prophages, transposons | 47 | 1.21 |
| Nucleotide transport and metabolism | 71 | 1.82 |
| Posttranslational modification, protein turnover, chaperones | 178 | 4.57 |
| RNA processing and modification | 1 | 0.03 |
| Replication, recombination, and repair | 145 | 3.72 |
| Secondary metabolites biosynthesis, transport, and catabolism | 119 | 3.05 |
| Signal transduction mechanisms | 365 | 9.36 |
| Transcription | 286 | 7.34 |
| Translation, ribosomal structure, and biogenesis | 268 | 6.88 |
| Not in COG | 1130 | 25.51 |
