# Supplementary material for: Chemical and genomic characterization of a potential probiotic treatment for stony coral tissue loss disease
Source: Commun Biol. 2023 Apr 6;6:248. doi: 10.1038/s42003-023-04590-y (PMC10079959; doi:10.1038/s42003-023-04590-y)
Supplement: Supplementary file 3 — Reporting Summary [file 42003_2023_4590_MOESM3_ESM.pdf]

nature portfolio

Corresponding author(s): Blake Ushijima (ushijimab@uncw.edu)
Valerie Paul (paul@si.edu)

Last updated by author(s): Jan 23, 2023

Reporting Summary

Nature Portfolio wishes to improve the reproducibility of the work that we publish. This form provides structure for consistency and transparency in reporting. For further information on Nature Portfolio policies, see our Editorial Policies and the Editorial Policy Checklist.

Please do not complete any field with "not applicable" or n/a. Refer to the help text for what text to use if an item is not relevant to your study.
For final submission: please carefully check your responses for accuracy; you will not be able to make changes later.

Statistics

For all statistical analyses, confirm that the following items are present in the figure legend, table legend, main text, or Methods section.

n/a	Confirmed	
☐	☒	The exact sample size (n) for each experimental group/condition, given as a discrete number and unit of measurement
☐	☒	A statement on whether measurements were taken from distinct samples or whether the same sample was measured repeatedly
☐	☒	The statistical test(s) used AND whether they are one- or two-sided<br>*Only common tests should be described solely by name; describe more complex techniques in the Methods section.*
☒	☐	A description of all covariates tested
☐	☒	A description of any assumptions or corrections, such as tests of normality and adjustment for multiple comparisons
☐	☒	A full description of the statistical parameters including central tendency (e.g. means) or other basic estimates (e.g. regression coefficient) AND variation (e.g. standard deviation) or associated estimates of uncertainty (e.g. confidence intervals)
☐	☒	For null hypothesis testing, the test statistic (e.g. F, t, r) with confidence intervals, effect sizes, degrees of freedom and P value noted<br>*Give P values as exact values whenever suitable.*
☒	☐	For Bayesian analysis, information on the choice of priors and Markov chain Monte Carlo settings
☒	☐	For hierarchical and complex designs, identification of the appropriate level for tests and full reporting of outcomes
☒	☐	Estimates of effect sizes (e.g. Cohen's d, Pearson's r), indicating how they were calculated

Our web collection on statistics for biologists contains articles on many of the points above.

Software and code

Policy information about availability of computer code

Data collection	Software used for the assembly and analysis of genomes included illumina-utils v. 2.3, cutadapt v. 1.8.1, SPAdes v. 3.13.0, antiSMASH v. 5.1.2, MiGA online, enveomics toolbox, NCBI BLAST+ v. 2.10.1, and MEGAX. R scripts for the analysis and plotting of ddPCR and 16S amplicon data are publicly available through https://github.com/meyermicrobiolab/McH1-7_Probiotics_Tank_Trials.
Data analysis	Software used for the assembly and analysis of genomes included illumina-utils v. 2.3, cutadapt v. 1.8.1, SPAdes v. 3.13.0, antiSMASH v. 5.1.2, MiGA online, enveomics toolbox, NCBI BLAST+ v. 2.10.1, and MEGAX. R scripts for the analysis and plotting of ddPCR and 16S amplicon data are publicly available through https://github.com/meyermicrobiolab/McH1-7_Probiotics_Tank_Trials.

For manuscripts utilizing custom algorithms or software that are central to the research but not yet described in published literature, software must be made available to editors and reviewers. We strongly encourage code deposition in a community repository (e.g. GitHub). See the Nature Portfolio guidelines for submitting code & software for further information.

Data

Policy information about availability of data

All manuscripts must include a data availability statement. This statement should provide the following information, where applicable:

- Accession codes, unique identifiers, or web links for publicly available datasets
- A description of any restrictions on data availability
- For clinical datasets or third party data, please ensure that the statement adheres to our policy

The sequencing reads for the 16S rRNA gene amplicons are available in NCBI under BioProject PRJNA801145. The sequencing reads and genome assembly for Pseudoalteromonas sp. strain McH1-7 are available in NCBI under BioProject PRJNA639770. All tissue measurement data associated with live coral experiments are publicly available on the Figshare database and can be accessed at https://figshare.com/s/18f60a82d908192c4014.

Human research participants

Policy information about studies involving human research participants and Sex and Gender in Research.

Reporting on sex and gender	This information has not been collected because human research participants were used in this study.
Population characteristics	This information has not been collected because human research participants were used in this study.
Recruitment	This information has not been collected because human research participants were used in this study.
Ethics oversight	No approval was needed because human research participants were used in this study.

Note that full information on the approval of the study protocol must also be provided in the manuscript.

Field-specific reporting

Please select the one below that is the best fit for your research. If you are not sure, read the appropriate sections before making your selection.

☒ Life sciences ☐ Behavioural & social sciences ☐ Ecological, evolutionary & environmental sciences

Life sciences study design

All studies must disclose on these points even when the disclosure is negative.

Sample size	Sample size was based on available coral, which is an incredibly limited resource. Statistical significance was calculated using the the statistical tests described in the paper once every block of data was gathered.
Data exclusions	The only data that was excluded was during probiotic effectiveness tests. If the control (untreated) specimens did not appear to have active disease (i.e. no disease progression) then that specimen and the paired experimental specimen were removed from the experiment.
Replication	Disease progression on coral fragments were analyzed using two different metrics, which were based on mortality rate and disease lesion progression, to ensure replication was sufficient. Replication was limited by number of corals available.
Randomization	Experimental groups (experimental blocks) were determined based on coral availability. When corals were available, experiments would be set up. Randomization was not possible due to the limited space and biosecurity associated with running experiments with diseased corals. Since each control and experimental pair were collected from the same coral colony (same genotype) and had the same disease lesion, randomizing all of the experimental was not considered necessary or feasible.
Blinding	Blinding was not possible for treatments that were inoculated with a probiotic or not. The probiotic made the water cloudy and the treatment was obvious.

Reporting for specific materials, systems and methods

We require information from authors about some types of materials, experimental systems and methods used in many studies. Here, indicate whether each material, system or method listed is relevant to your study. If you are not sure if a list item applies to your research, read the appropriate section before selecting a response.

Materials & experimental systems

n/a	Involved in the study
☒	☐ Antibodies
☒	☐ Eukaryotic cell lines
☒	☐ Palaeontology and archaeology
☐	☒ Animals and other organisms
☒	☐ Clinical data
☒	☐ Dual use research of concern

Methods

n/a	Involved in the study
☒	☐ ChIP-seq
☒	☐ Flow cytometry
☒	☐ MRI-based neuroimaging

Animals and other research organisms

Policy information about studies involving animals; ARRIVE guidelines recommended for reporting animal research, and Sex and Gender in Research

Laboratory animals	Laboratory animals were not used in this study.
Wild animals	The corals Montasraea cavernosa and Orbicella faveolata were used in this study. Their sex was not known. Orbicella faveolata are hermaphrodites. Determining age was not possible for these colonial organisms. Corals were collected with a hammer and chisel, wrapped in wet bubble wrap, and transported in coolers to coral facilities. All specimens were maintained in sterilized seawater. Corals were further fragmented using a diamond blade masonry saw.
Reporting on sex	This information was not collected. The corals Montasraea cavernosa and Orbicella faveolata were used in this study. Their sex was not known. Orbicella faveolata are hermaphrodites.
Field-collected samples	Portions of diseased colonies were collected while scuba diving using a hammer and chisel from various locations around Broward County and the Florida Keys in compliance with the appropriate permits from the Florida Fish and Wildlife Conservation Commission and Florida Keys National Marine Sanctuary, respectively. To reduce the chance of collecting corals with the same genotype, colonies at least 5 m apart were selected for use. All healthy corals used in the experiments described here were received from the NOAA Key West coral nursery. All corals collected from the field or from the nursery were transported to the Smithsonian Marine Station (SMS) facility in Fort Pierce, FL after being wrapped in plastic bubble wrap moistened with seawater and then placed in a cooler. Upon arrival at the SMS facility, each fragment was gently rinsed with FSW to remove excess mucus and loose debris. Healthy and diseased corals were handled and maintained in separate areas of the facility 10. Briefly, all corals were maintained in temperature-controlled systems and were initially maintained at the same temperature as the collection site but were then slowly adjusted to 28 °C (a maximum 0.5°C change per day) before use in experiments. Diseased corals were held in buckets with 13 L of FSW and a weighted airline in outdoor systems under ambient light conditions with a clear plastic canopy and one layer of shade cloth that blocked approximately 50% of the sunlight. Partial water changes (approximately 50% water exchange) were conducted at different intervals depending on the experiment (see below). Sterilized plastic scoops were used for all water changes. Scoops were rinsed and scrubbed in a 10% calcium hypochlorite solution, rinsed with freshwater several times, and then left to dry for at least 24 h before each use. Apparently healthy corals were kept in an indoor facility within multiple large recirculating water table systems each holding approximately 570 L of FSW. A row of six blue-white 30 cm2 LED panels (HQPR) were fixed above each table providing 150 to 250 µmol photons m-2 sec-1 for captive corals.
Ethics oversight	No ethical approval was needed for this work with corals (invertebrates). The proper collection permits were obtained before any specimens were obtained previous to sample collection.

Note that full information on the approval of the study protocol must also be provided in the manuscript.

This checklist template is licensed under a Creative Commons Attribution 4.0 International License, which permits use, sharing, adaptation, distribution and reproduction in any medium or format, as long as you give appropriate credit to the original author(s) and the source, provide a link to the Creative Commons license, and indicate if changes were made. The images or other third party material in this article are included in the article's Creative Commons license, unless indicated otherwise in a credit line to the material. If material is not included in the article's Creative Commons license and your intended use is not permitted by statutory regulation or exceeds the permitted use, you will need to obtain permission directly from the copyright holder. To view a copy of this license, visit http://creativecommons.org/licenses/by/4.0/
